# Optimal projection method determination by Logdet Divergence and perturbed von-Neumann Divergence

**DOI:** 10.1186/s12918-017-0479-0

**Published:** 2017-12-14

**Authors:** Hao Jiang, Wai-Ki Ching, Yushan Qiu, Xiao-Qing Cheng

**Affiliations:** 1 Department of Mathematics, School of Information, Renmin University of China, No.59 Zhong Guan Cun Street, Hai Dian District, Beijing, 100872 China; 2 Department of Mathematics, The University of Hong Kong, Pokfulam Road, Hong Kong, Hong Kong; 3 College of Mathematics and Statistics, Shenzhen University, Nanhai Avenue 3688, Shenzhen, 518060 China; 4 School of Mathematics and Statistics, Xi’An Jiaotong University, No.28 West Xianning Road, Xi’An, 710049 China

**Keywords:** SVM, Indefinite kernel, Projection method, Bregman matrix divergence

## Abstract

**Background:**

Positive semi-definiteness is a critical property in kernel methods for Support Vector Machine (SVM) by which efficient solutions can be guaranteed through convex quadratic programming. However, a lot of similarity functions in applications do not produce positive semi-definite kernels.

**Methods:**

We propose projection method by constructing projection matrix on indefinite kernels. As a generalization of the spectrum method (denoising method and flipping method), the projection method shows better or comparable performance comparing to the corresponding indefinite kernel methods on a number of real world data sets. Under the Bregman matrix divergence theory, we can find suggested optimal *λ* in projection method using unconstrained optimization in kernel learning. In this paper we focus on optimal *λ* determination, in the pursuit of precise optimal *λ* determination method in unconstrained optimization framework. We developed a perturbed von-Neumann divergence to measure kernel relationships.

**Results:**

We compared optimal *λ* determination with Logdet Divergence and perturbed von-Neumann Divergence, aiming at finding better *λ* in projection method. Results on a number of real world data sets show that projection method with optimal *λ* by Logdet divergence demonstrate near optimal performance. And the perturbed von-Neumann Divergence can help determine a relatively better optimal projection method.

**Conclusions:**

Projection method ia easy to use for dealing with indefinite kernels. And the parameter embedded in the method can be determined through unconstrained optimization under Bregman matrix divergence theory. This may provide a new way in kernel SVMs for varied objectives.

**Electronic supplementary material:**

The online version of this article (doi:10.1186/s12918-017-0479-0) contains supplementary material, which is available to authorized users.

## Background

Support vector machines (SVMs), a supervised machine learning technique, have been introduced by Vapnik [1, 2]. In machine learning area, SVMs [3] are traditionally considered as one of the best algorithms in terms of structural risk minimization. Kernels in SVM work by data embedding in high dimensional feature space and one can construct an optimal separating hyperplane in this space [4]. Furthermore, kernel methods have wide applications in the field of bioinformatics. Authors in [5] have proposed incremental kernel ridge regression to predict soft tissue deformations after CMF surgery. In [6], researchers utilized the kernel-based linear discriminant analysis (LDA) method to address the problem of automatically tuning multiple kernel parameters. In order to address the nonlinear problem of nonnegative matrix factorization (NMF) and the semi-nonnegative problem of the existing kernel NMF methods, authors in [7] develop the nonlinear NMF based on a self-constructed Mercer kernel which preserves the nonnegative constraints on both bases and coefficients in kernel feature space. Positive Semi-Definiteness (PSD) is crucial [8] for a kernel matrix in SVMs, which is required to guarantee the existence of a Reproducing Kernel Hilbert Space (RKHS). In RKHS, one can formulate a convex optimization problem to obtain an optimal solution. Sometimes however, similarity matrices generated for practical use cannot ensure such a PSD property. For example, in evaluation of pair-wise similarity between DNA and protein sequences, popular functions like BLAST and Dynamic Time Warping generate indefinite kernel matrices [9–11]. The generalized histogram intersection kernel that is conditionally positive definite is not usually positive semi-definite [12]. Hyperbolic tangent kernels [13, 14] suitable for practice sometimes are indefinite as well. As far as we know, it is still not very clear how to effectively deal with indefinite kernels in the SVM framework. Training indefinite SVMs therefore becomes a challenging optimization problem since convex solutions are no longer valid for standard SVMs in this learning scenario [15].

To deal with indefinite kernel, a number of methods have been proposed in the literature [16]. Representatives in previous studies tackled such problem by altering the spectrum of an indefinite kernel matrix so as to create a PSD one. Authors in [17] developed the denoising method which deems negative eigenvalues as noise and replaces them with zero. The flipping method is another effective method for transforming indefinite kernel into PSD one by changing the sign of negative eigenvalues [18]. Authors in [19] proposed the diffusion method which considers the data distribution and replaces the eigenvalues with exponential form. The shifting method, i.e., shifts eigenvalues by introducing new parameters to ensure all the eigenvalues are nonnegative [20]. Authors in [13] developed a method in order to find stationary points under a non-convex dual formulation of SVMs with sigmoid kernels. Authors considered indefinite kernel learning as a minimization problem in a pseudo-Euclidean space in [21]. In [22], a max-min optimization problem is further proposed so as to find a proxy kernel for the indefinite kernel. Based on confidence function, a simple generalization of SVMs is suggested by Guo and Schuurmans [23]. Kernel principal component analysis is developed as a kernel transformation method to deal with indefinite kernels [24].

In this paper, we develop a superior and effective method, i.e., projection method, to convert an indefinite kernel into a PSD one. Compared with the existing methods, our proposed one is much more flexible and comprehensive. One can easily obtain different type of methods such as flipping or denoising method by varying its parameters. Furthermore, our suggested *λ* under Logdet Divergence and perturbed von-Neumann Divergence can always yield near optimal performance, which can be regarded as a good choice for dealing with indefinite kernels. Besides, our suggested projection matrix also has certain special mathematical properties. Furthermore, the connection between spectrum method and projection method can be investigated through analysis on eigenvalues.

The rest of the paper is organized as follows. Firstly, we present the projection method and also the associated theorem. Then we propose the optimal *λ* determination in the projection matrix under unconstrained optimization framework. After that, we apply two indefinite kernels on some real world data sets which range from cancer prediction to glycan classification. And we also validate the suggested optimal *λ* with the experimental data. Discussions of the experimental validation on the suggested optimal *λ* under Logdet Divergence and perturbed von-Neumann Divergence are followed. Finally, in the last section, we give the concluding remarks with possible future work.

## Methods

Assume ${(\mathbf {X}_{i},y_{i})}_{i=1}^{n}$, where **X**_*i*_∈**R** ^*p*^, and *y*_*i*_∈{1,−1} are a given list of labeled patterns. And function *k* defined as *k*:*χ*×*χ*→**R** can be regarded as a kernel function where *χ* represents the input space. A kernel induced by the kernel function is defined by

1 $$  K(i,j)=k(\mathbf{X}_{i},\mathbf{X}_{j}), \qquad i,j \in \{1,2,\ldots,n\}  $$

And according to Mercer’s theorem, a valid kernel should be positive semi-definite. Thus, to deal with invalid kernels, kernel transformation strategy is increasingly popular. In the case of non-positive semi-definite kernel *K*, we may decompose it into this form *K*=*P*·*D*·*P* ^′^. Where *D* is a diagonal matrix and not all the diagonal entries are non-negative, *P* is orthonormal matrix with the *j*th column corresponding to the eigenvector for *j*th eigenvalue in *D* and *P* ^′^ represents the transpose of matrix *P*. Eigenvalue transform is the representative method in kernel transformation [17–20].

In the following, we present our suggested projection method for transforming an indefinite kernel to a PSD one.

### **Lemma 1**

There exists an *n*×*m* (*m*<*n*) matrix *B* satisfying $B^{\prime }B=I_{m}$ such that $(I_{n}-\lambda BB^{\prime })$ has 1−*λ* and 1 as its eigenvalues, the multiplicities for whom are *m* and *n*−*m* respectively. Besides, it shares the same set of eigenvectors with *K*.

### *Proof*

Consider that *K* is a real and symmetric matrix, we decompose it as *K*=*P*·*D*·*P* ^′^ where $P=[\vec {p}_{1},\vec {p}_{2},\ldots,\vec {p}_{n}]$ and *D*=diag[*d*_1_,*d*_2_,…,*d*_*n*_] is a diagonal matrix with the diagonal elements *d*_*i*_,*i*=1,2,…,*n*. W.L.O.G, we may assume all the eigenvalues are sorted in ascending order. We further assume the positive inertia index is *l* and the negative inertia index is *m*.

Denote $B=[\vec {p}_{1},\vec {p}_{2},\ldots,\vec {p}_{m}]$, we have *B* ^′^ *B*=*I*_*m*_, since $\vec {p}_{i},i=1,2,\ldots,n$ are orthogonal eigenvectors. Then we have

2 $$  (I_{n}-\lambda BB')\vec{p}_{i}=\left\{ \begin{aligned} (1-\lambda)\vec{p}_{i}, \quad \quad i\in\{1,2,\ldots,m\}\\ \vec{p}_{i}, \quad i\in\{m+1,m+2,\ldots,n\}\\ \end{aligned} \right.  $$

Thus, it has 1 and (1−*λ*) as its eigenvalues, and the multiplicity for (1−*λ*) is *m* and for 1 the multiplicity is *n*−*m*. Furthermore, the eigenvectors of (*I*_*n*_−*λ* *B* *B* ^′^) are exactly the same as the kernel *K*. □

### **Theorem 1**

Let *K* be an *n*×*n* real symmetric matrix which is indefinite. Then there exists an *n*×*m* (*m*<*n*) matrix *B* satisfying *B* ^′^ *B*=*I*_*m*_ such that (*I*_*n*_−*λ* *B* *B* ^′^)*K* is a positive semi-definite kernel where *λ*≥1 is a regularization parameter.

### *Proof*

Denote $B=[\vec {p}_{1},\vec {p}_{2},\ldots,\vec {p}_{m}]$, where the definitions of $\vec {p}_{i}, i=1,2,\ldots,m$ are the same as denoted in Lemma 1. By Eq. (2), we have

3 $$\begin{array}{@{}rcl@{}}  \begin{array}{lll} &&(I_{n}-\lambda BB')K\\&=&P\text{diag}\{1-\lambda,\ldots,1-\lambda,1,\ldots,1\}P'\cdot\\ & &P\text{diag}\{d_{1},d_{2},\ldots,d_{n}\}P'\\ &=&P\text{diag}\{(1-\lambda)d_{1}, \ldots,(1-\lambda)d_{m},\\& &\underbrace{0,\ldots,0}_{n-m-l},d_{n-l+1},\ldots,d_{n}\}P'. \end{array} \end{array} $$

Since *λ*≥1, we have (1−*λ*)*d*_*i*_≥0 for 1≤*i*≤*m*. This will guarantee the kernel matrix (*I*_*n*_−*λ* *B* *B* ^′^)*K* is positive semi-definite. □

In particular, we get denoising method by letting *λ*=1 according to Eq. (3). And flipping method is the particular case of Projection method when *λ*=2.

### Optimal *λ* determination

Considering that *λ* is a embedded parameter in the projection method, it is necessary to study optimal *λ* determination which can demonstrate excellent prediction power for *λ*>0. To this end, we begin with the definition of Bregman matrix divergence [25].

#### **Definition 1**

{Bregman Matrix Divergences}The Bregman Matrix Divergence of *K* is defined as follows:

$$D_{\phi}(K,K_{0})=\phi(K)-\phi(K_{0})-\text{tr}(\nabla\phi(K_{0}))'(K-K_{0}). $$

Here *ϕ*(*K*)is a strictly convex differentiable function of *K* and tr(*K*) means the trace of matrix *K*.

A number of matrix divergences [25, 26] exist in the literature.

1. Mahalanobis Divergence(*p*=2):
  4 $$  D_{\phi}(K,K_{0})=\text{tr}\left(K^{2}-2{KK}_{0}+K_{0}^{2}\right).  $$
2. Frobenius Divergence$\left (\phi (K) = \|K\|_{F}^{2}\right)$:
  5 $$  D_{\phi}(K,K_{0})=\|K-K_{0}\|_{F}^{2}.  $$
3. von-Neumann Divergence(*ϕ*(*K*)=tr(*K* log(*K*)−*K*)):
  6 $$  D_{\phi}(K,K_{0})=\text{tr}(K\log K- K \log K_{0}- K + K_{0}).  $$
4. LogDet Divergence(*ϕ*(*K*)=− log det(*K*)):
  7 $$ {}D_{\phi}(K,K_{0})=\text{tr}\left({KK}_{0}^{-1}\right)-\log \det\left({KK}_{0}^{-1}\right)-n.  $$

Inspired by the work in [27] where authors proposed a framework of kernel learning [28] by unconstrained optimization, we re-formulate the problem as a kernel learning one in a similar manner. The optimal *λ* can be obtained by minimization of $D_{\phi }(\tilde {K},K)$ where $\tilde {K}$ is the optimal PSD kernel which is close to original kernel *K* in terms of divergence. Noting that

$$K = \sum\limits_{i=1}^{n} d_{i}\vec{p}_{i}\vec{p}_{i}',\quad \tilde{K}=\sum\limits_{i=1}^{m} (1-\lambda)d_{i}\vec{p}_{i}\vec{p}_{i}'+\sum\limits_{i=m+1}^{n}d_{i}\vec{p}_{i}\vec{p}_{i}' $$

then the minimization problem can be equivalently transformed to the following:

For Mahalanobis Divergence, by Lemma, we know that *K* *K*_0_=*K*_0_ *K* as they share the same set of eigenvectors. Therefore, the minimization problem can be expressed as

8 $$  \min_{\lambda}\lambda^{2} \text{tr}(BB'K)^{2}  $$

The optimal *λ* can be quickly obtained as 0.

For Frobenius Divergence, the minimization problem in finding optimal *λ* as derived from Eq. (5) is

9 $$  \min_{\lambda}\lambda \|BB'K\|_{F}^{2}  $$

It is easy to see that the optimal *λ* is 0.

For von Neumann Divergence, we can deduce the minimization problem to be

10 $$  \min_{\lambda} \sum\limits_{i=1}^{m}d_{i}((1-\lambda)\text{log}(1-\lambda)+\lambda)  $$

Applying differentiation to Eq. (10), we obtain the optimal value of *λ*=0.

The optimal *λ*=0 does not make any perturbation to the original kernel matrix which is not reasonable. Hence we focus on LogDet Divergence [27], the optimal *λ* can be determined through the following formula

11 $$  -\frac{m}{1-\lambda}-\sum_{i=1}^{m} d_{i} \text{tr}(K^{-1} \vec{p}_{i}\vec{p}_{i}')=0.  $$

Considering that the calculation involves inverse of matrix *K*, where *K* is not necessarily positive definite, we use pseudo inverse instead. Thus, the final theoretical optimal *λ* becomes:

12 $$  \lambda_{\text{opt}}=1+\frac{m}{\sum_{i=1}^{m}d_{i} \text{tr}(K^{-1}\vec{p}_{i}\vec{p}_{i}')}.  $$

## Results

### Materials

In order to experimentally evaluate our method, we adopted a number of life science data sets satisfying requirements that the generated kernels are indefinite, where most of them are cancer related data sets. Three data sets are obtained from libsvm data sets [29]. One of the data sets is sonar data, there are 208 data instances where 97 are positive and 111 are negative. The Live disorder data set has 345 data instances, of which 145 are positive and 200 are negative. Breast Cancer data set has 683 data instances in total, 444 are negative and 239 are positive, and the number of attributes is 10. Another two datasets pertain to cystic fibrosis and leukemia. Within the cystic fibrosis data set, there are 177 glycan structures in total, containing 89 glycans related to cystic fibrosis, 107 related to respiratory mucin and 101 related to bronchial mucin. For leukemia related data set, 355 structures are included,originating from four human blood components: leukemic cells, erythrocytes, serum and plasma, containing 162, 111, 85 and 73 examples respectively. All the glycan structures are retrieved from the KEGG/GLYCAN database [30], where annotations are retrieved from CarbBank/CCSD database [31]. If the glycan data set contains *N* glycans { *g*_1_,*g*_2_,⋯,*g*_*N*_}, we denote the set of all *q*-grams existing in these *N* glycans to be a *q*-gram set: ${\Phi }_{q}=\{{\phi }_{q}^{1},{\phi }_{q}^{2},\cdots, {\phi }_{q}^{n_{q}}\}$. For a specific glycan *g*_*i*_ in the data set, *q*-gram representation is a column vector $ x_{i}^{q}=[x_{1i}^{q},x_{2i}^{q}, \cdots, x_{n_{q}i}^{q}]^{T} $ where $x_{li}^{q}$ means the number of *l*th *q*-gram in the glycan *g*_*i*_. The number of attributes within the dataset depends on the value of *q* (*q*=1 to 9), where we have 9 datasets derived from cystic fibrosis data and leukemia data respectively. The last data set is about lung cancer and it is obtained from NCBI(National Center of Biotechnology Information) GEO(Gene Expression Omnibus) [32]. Affymetrix Human Genome U133 Plus 2.0 Array experiments were carried out in a set of 91 non-small cell lung cancer (NSCLC) samples, containing 46 tumors and 45 controls. Detailed information on the data sets can be found in Table 1.

**Table 1:** Data set information

Data set	Number of instances	Number of attributes
Sonar	208	60
Live disorder	345	6
Breast cancer	680	10
Cystic fibrosis	177	Depends on q
Leukemia	355	Depends on q
Lung cancer	91	54675

**Fig. 1:**
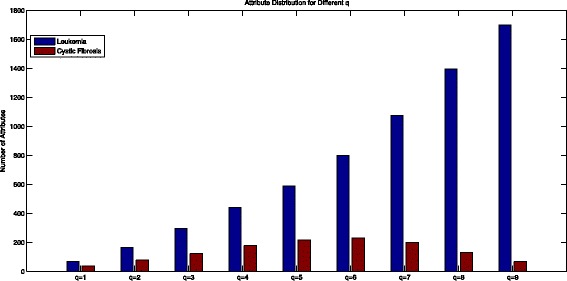
Attribute distribution for different values of q in leukemia data and cystic fibrosis data

Attribute Distribution Information for different *q* is provided in Fig. 1 for Leukemia Data and Cystic Fibrosis Data. We can see that the number of attributes in Leukemia Data is increasing with the increment of *q* while it is not the case in Cystic Fibrosis Data. In Cystic Fibrosis Data set, the number of the attributes firstly increases then decreases with the increment of *q*. The possible reason is that the glycan structures in Leukemia Data set is more complicated than that in Cystic Fibrosis Data set.

### Experiments

We perform the experiments in 5-fold cross-validation setting and measure the performance of models with the Area Under Curve (AUC). AUC (calculated as the area under the ROC curve) is commonly used for model evaluation. We measure the averaged AUC values for the considered methods through 10 times 5-fold cross-validations. Here we introduce two kernels: the Generalized Histogram Intersection (GHI) kernel [12] and the cosine kernel for illustration purpose. These two kernels in most cases are indefinite (shown in Additional file 1: Table SI), both of which have not been used in biological applications like glycan classification or cancer prediction.

GHI kernel is frequently used in image classification and the definition is as follows:

$$k(\mathbf{X}_{j},\mathbf{X}_{k}) =\sum\limits_{i=1}^{p} \min (|\mathbf{X}_{ji}|^{\alpha},|\mathbf{X}_{ki}|^{\beta}) $$

where **X**_*j*_ represents data vector and **X**_*ji*_ represents the *i*th element of **X**_*j*_,*j*=1,2,…,*n*.

When *α*=*β*, the kernel can be proved to be a positive semi-definite matrix. Experimental results in Table SI (in Additional file 1) also show consistence with the statement, as the minimal eigenvalue in GHI kernel is 0 when *α*=*β*. We in experimental settings use different values of *α*,*β*∈{1,2,3},*α*≠*β* to evaluate the performance of our proposed projection method.

The cosine kernel function is defined by:

$$k(\mathbf{X}_{j},\mathbf{X}_{k}) = \cos(||\mathbf{X}_{j}-\mathbf{X}_{k}||) $$

which is different from the usual definition of cosine similarity: $\frac {\mathbf {X}_{j}'\mathbf {X}_{k}}{||\mathbf {X}_{j}||\cdot ||\mathbf {X}_{k}||}$. The reason we did not consider the usual definition is that the corresponding kernel matrix generated from this function is positive semi-definite, which fails to satisfy our requirements.

#### Experiments on GHI kernel

The performance of Projection method and original GHI Kernel SVM was summarized in Tables 2, 3, and 4. Values marked in bold face represent best performance and no marks are made when both methods showing comparable performance. When *α* differs from *β*, GHI kernel is indefinite (see Additional file 1: Table SI), we can see that Projection Method outperforms GHI Kernel method.

**Table 2:** Averaged AUC values (%) of projection method and GHI kernel using sonar data, live disorder data, breast cancer data and NSCLC data

Data sets	Parameters	Projection method	GHI kernel
Sonar	*α*=1,*β*=1	82.87 ± 0.99	82.87 ± 0.99
	*α*=1,*β*=2	**81.47** ± **0.99**	53.42 ± 4.94
	*α*=1,*β*=3	**84.02** ± **1.19**	54.10 ± 4.92
	*α*=2,*β*=2	84.29 ± 1.54	84.29 ± 1.54
	*α*=2,*β*=3	**84.31** ± **1.56**	83.06 ± 2.04
	*α*=3,*β*=3	83.62 ± 1.17	83.62 ± 1.17
Live	*α*=1,*β*=1	82.87 ± 0.99	82.87 ± 0.99
	*α*=1,*β*=2	**81.47** ± **0.99**	53.42 ± 4.94
	*α*=1,*β*=3	**84.02** ± **1.19**	54.10 ± 4.92
	*α*=2,*β*=2	84.29 ± 1.54	84.29 ± 1.54
	*α*=2,*β*=3	**84.31** ± **1.56**	83.06 ± 2.04
	*α*=3,*β*=3	83.62 ± 1.17	83.62 ± 1.17
Breast	*α*=1,*β*=1	96.73 ± 0.11	96.73 ± 0.11
	*α*=1,*β*=2	**97.06** ± **0.01**	90.12 ± 4.78
	*α*=1,*β*=3	**97.01** ± **0.01**	75.61 ± 7.44
	*α*=2,*β*=2	96.71 ± 0.11	96.71 ± 0.11
	*α*=2,*β*=3	96.92 ± 0.01	96.96 ± 0.01
	*α*=3,*β*=3	96.63 ± 0.10	96.63 ± 0.10
NSCLC	*α*=1,*β*=1	100 ± 0	100 ± 0
	*α*=1,*β*=2	**99.72** ± **0.01**	64.07 ± 7.42
	*α*=1,*β*=3	**61.46** ± **1.57**	51.47 ± 5.53
	*α*=2,*β*=2	100 ± 0	100 ± 0
	*α*=2,*β*=3	**99.99** ± **0**	73.07 ± 8.17
	*α*=3,*β*=3	100 ± 0	100 ± 0

Bold face represents best performance, and no marks are made if two methods show comparable performance

**Table 3:** Averaged AUC values (%) of projection method and GHI kernel using cystic fibrosis data

Parameters	Projection method(*q*=1)	GHI(*q*=1)	Projection method(*q*=2)	GHI(*q*=2)
*α*=1,*β*=1	78.57 ± 1.75	78.57 ± 1.75	81.32 ± 1.25	81.32 ± 1.25
*α*=1,*β*=2	78.94 ± 1.86	78.94 ± 1.86	81.74 ± 1.60	81.74 ± 1.60
*α*=1,*β*=3	78.64 ± 1.01	78.63 ± 1.01	80.82 ± 1.30	80.82 ± 1.29
*α*=2,*β*=2	79.33 ± 1.42	79.33 ± 1.41	80.53 ± 1.72	80.53 ± 1.72
*α*=2,*β*=3	79.32 ± 1.19	79.32 ± 1.19	81.06 ± 1.37	81.06 ± 1.36
*α*=3,*β*=3	78.14 ± 1.11	78.13 ± 1.11	80.79 ± 1.12	80.78 ± 1.12
Parameters	Projection method(*q*=3)	GHI(*q*=3)	Projection method(*q*=4)	GHI(*q*=4)
*α*=1,*β*=1	80.77 ± 1.44	80.76 ± 1.44	83.10 ± 2.10	83.09 ± 2.10
*α*=1,*β*=2	80.98 ± 1.81	80.97 ± 1.81	82.11 ± 1.77	82.13 ± 1.77
*α*=1,*β*=3	81.20 ± 1.95	81.19 ± 1.94	83.54 ± 1.46	83.51 ± 1.48
*α*=2,*β*=2	81.32 ± 1.26	81.30 ± 1.27	82.75 ± 2.14	82.79 ± 2.15
*α*=2,*β*=3	81.10 ± 1.10	81.09 ± 1.11	83.62 ± 1.61	83.65 ± 1.65
*α*=3,*β*=3	81.06 ± 1.39	81.04 ± 1.39	83.49 ± 0.77	83.56 ± 0.82
Parameters	Projection method(*q*=5)	GHI(*q*=5)	Projection method(*q*=6)	GHI(*q*=6)
*α*=1,*β*=1	74.03 ± 2.18	74.00 ± 2.18	72.30 ± 1.93	72.50 ± 1.87
*α*=1,*β*=2	71.67 ± 2.52	71.62 ± 2.58	73.62 ± 2.69	73.80 ± 2.70
*α*=1,*β*=3	74.77 ± 2.27	74.73 ± 2.28	71.94 ± 1.77	72.11 ± 1.65
*α*=2,*β*=2	73.73 ± 1.36	73.73 ± 1.38	71.49 ± 2.78	71.60 ± 2.84
*α*=2,*β*=3	72.62 ± 2.97	72.61 ± 2.92	72.81 ± 1.91	73.01 ± 1.92
*α*=3,*β*=3	75.23 ± 2.64	75.20 ± 2.55	73.53 ± 2.62	73.80 ± 2.67
Parameters	Projection method(*q*=7)	GHI(*q*=7)	Projection method(*q*=8)	GHI(*q*=8)
*α*=1,*β*=1	67.99 ± 2.78	67.60 ± 2.87	60.65 ± 4.20	60.90 ± 4.36
*α*=1,*β*=2	68.28 ± 3.51	67.89 ± 3.60	58.19 ± 3.72	58.33 ± 3.77
*α*=1,*β*=3	67.75 ± 2.20	67.25 ± 2.19	58.98 ± 3.67	59.28 ± 3.69
*α*=2,*β*=2	67.90 ± 3.11	67.23 ± 3.04	58.28 ± 4.20	58.34 ± 4.13
*α*=2,*β*=3	67.58 ± 2.91	66.96 ± 2.88	58.66 ± 2.40	58.86 ± 2.37
*α*=3,*β*=3	68.85 ± 2.28	68.44 ± 2.13	59.62 ± 3.34	59.77 ± 3.37
Parameters	Projection method(*q*=9)	GHI(*q*=9)		
*α*=1,*β*=1	53.25 ± 3.99	53.25 ± 3.99		
*α*=1,*β*=2	52.12 ± 4.28	52.12 ± 4.28		
*α*=1,*β*=3	52.54 ± 3.22	52.54 ± 3.22		
*α*=2,*β*=2	51.16 ± 2.37	51.16 ± 2.37		
*α*=2,*β*=3	51.62 ± 4.18	51.62 ± 4.18		
*α*=3,*β*=3	51.96 ± 5.01	51.96 ± 5.01

**Table 4:** Averaged AUC values (%) of projection method and GHI kernel using leukemia data

Parameters	Projection method(*q*=1)	GHI(*q*=1)	Projection method(*q*=2)	GHI(*q*=2)
*α*=1,*β*=1	93.68 ± 0.62	93.68 ± 0.62	95.90 ± 0.84	95.90 ± 0.84
*α*=1,*β*=2	**93.75** ± **0.59**	87.00 ± 4.10	**95.85** ± **0.41**	94.93 ± 0.81
*α*=1,*β*=3	**93.34** ± **0.91**	86.94 ± 3.37	**95.41** ± **0.64**	94.53 ± 0.89
*α*=2,*β*=2	93.33 ± 0.74	93.32 ± 0.74	95.61 ± 0.46	95.61 ± 0.46
*α*=2,*β*=3	93.31 ± 0.47	93.51 ± 0.46	95.17 ± 0.69	95.66 ± 0.85
*α*=3,*β*=3	93.54 ± 0.66	93.54 ± 0.66	95.77 ± 0.40	95.77 ± 0.40
Parameters	Projection method(*q*=3)	GHI(*q*=3)	Projection method(*q*=4)	GHI(*q*=4)
*α*=1,*β*=1	95.07 ± 0.64	95.08 ± 0.65	93.51 ± 0.54	93.54 ± 0.54
*α*=1,*β*=2	95.13 ± 0.46	95.10 ± 0.471	93.86 ± 0.77	93.88 ± 0.77
*α*=1,*β*=3	94.83 ± 0.41	94.77 ± 0.42	94.13 ± 0.52	94.15 ± 0.52
*α*=2,*β*=2	95.13 ± 0.53	95.13 ± 0.53	94.05 ± 0.49	94.06 ± 0.49
*α*=2,*β*=3	94.84 ± 0.67	94.85 ± 0.67	93.81 ± 0.69	93.82 ± 0.69
*α*=3,*β*=3	94.77 ± 0.61	94.77 ± 0.61	93.98 ± 0.36	93.99 ± 0.36
Parameters	Projection method(*q*=5)	GHI(*q*=5)	Projection method(*q*=6)	GHI(*q*=6)
*α*=1,*β*=1	93.40 ± 0.58	93.44 ± 0.58	93.23 ± 0.26	93.38 ± 0.26
*α*=1,*β*=2	93.12 ± 0.70	93.16 ± 0.70	93.07 ± 0.75	93.21 ± 0.74
*α*=1,*β*=3	93.20 ± 0.27	93.25 ± 0.28	93.05 ± 0.63	93.18 ± 0.63
*α*=2,*β*=2	93.61 ± 0.73	93.64 ± 0.74	93.21 ± 0.48	93.35 ± 0.48
*α*=2,*β*=3	93.78 ± 0.56	93.83 ± 0.56	93.26 ± 0.70	93.41 ± 0.72
*α*=3,*β*=3	93.71 ± 0.72	93.75 ± 0.73	93.38 ± 0.65	93.51 ± 0.67
Parameters	Projection method(*q*=7)	GHI(*q*=7)	Projection method(*q*=8)	GHI(*q*=8)
*α*=1,*β*=1	92.15 ± 0.68	92.37 ± 0.67	90.10 ± 0.71	90.36 ± 0.70
*α*=1,*β*=2	92.33 ± 0.57	92.53 ± 0.59	90.68 ± 1.14	90.92 ± 1.13
*α*=1,*β*=3	92.11 ± 0.86	92.31 ± 0.86	90.72 ± 0.73	90.96 ± 0.73
*α*=2,*β*=2	92.01 ± 0.50	92.23 ± 0.50	90.67 ± 1.06	90.93 ± 1.04
*α*=2,*β*=3	92.06 ± 0.45	92.27 ± 0.43	90.31 ± 0.90	90.53 ± 0.89
*α*=3,*β*=3	92.28 ± 0.71	92.48 ± 0.73	90.66 ± 0.65	90.92 ± 0.67
Parameters	Projection method(*q*=9)	GHI(*q*=9)		
*α*=1,*β*=1	88.92 ± 0.59	89.20 ± 0.62		
*α*=1,*β*=2	89.61 ± 0.62	89.86 ± 0.63		
*α*=1,*β*=3	89.33 ± 0.68	89.60 ± 0.67		
*α*=2,*β*=2	89.54 ± 0.96	89.80 ± 0.96		
*α*=2,*β*=3	88.57 ± 0.67	88.82 ± 0.68		
*α*=3,*β*=3	88.56 ± 0.63	88.84 ± 0.63		

Bold face represents best performance for leukemia data in the compared two methods: Projection method and GHI Kernel method, and no marks are made if two methods show comparable performance

The performance for sonar data set is reported in Table 2. For example, when *α*=1,*β*=2, Projection Method shows the averaged AUC value 81.47% with standard deviation 0.99% while in GHI kernel method the averaged AUC value is 53.42% with standard deviation 4.94%. When (*α*,*β*)=(1,3), Projection Method shows 84.02% in the averaged AUC value, with standard deviation 1.19%. However, the averaged AUC value for GHI kernel method is only 54.10% with standard deviation 4.92%. When (*α*,*β*)=(2,3), the averaged AUC value for Projection Method is 84.31%, larger than the averaged AUC value for GHI Method 83.06%. The standard deviation in Projection Method is 1.56%, while in GHI kernel method standard deviation is 2.04%. This implies that Projection method is more powerful and stable compared to original GHI kernel method.

For live disorder data set, we can see from Table 2 that the Projection method is significantly better performance than the GHI kernel method when *α*≠*β*. The best performance of GHI kernel when indefinite achieves around 60% in AUC value which is not satisfying. When *α*=*β*, both methods show comparable performance.

For breast cancer data set, results in Table 2 indicate that when *α*=1,*β*=2 and *α*=1,*β*=3, the Projection method is clearly superior to the GHI kernel method except for *α*=2,*β*=3 where comparable performance is detected in both methods. This illustrates the fact that indefinite kernels sometimes can also perform well. However, the superiority of projection method over the original GHI kernel method is clearly shown in this data set.

In cystic fibrosis data set, we get 9 different comparison results when values of *q* vary from 1 to 9 as shown in Table 3. There is no clear difference between Projection method and GHI kernel, as GHI kernel is positive semi-definite for almost all considered pairs of *α* and *β* (see Additional file 1: Table SI for reference). The only 2 cases when GHI kernel indefinite are *α*=1,*β*=2 and *α*=1,*β*=3 for q = 1, and the minimal eigenvalue for the generated GHI kernel in these 2 cases is only -0.08, quite close to 0, demonstrating that the generated kernel is almost positive semi-definite.

Results for Leukemia Data are summarized in Table 4. Similar to the results in cystic fibrosis data, projection method and GHI kernel method show similar performance in most of the cases for *q* from 1 to 9. From Additional file 1: Table SI we can see that, GHI kernel is indefinite when *α*≠*β* for *q*=1,2,3. When *q*=1,2, Projection method is better than GHI kernel method for *α*=1,*β*=2 and *α*=1,*β*=3; However, GHI kernel method is comparable to Projection method for *α*=2,*β*=3.

Some interesting results can be found for NSCLC data as shown in Table 2. Projection method and GHI kernel method show exact performance when *α*=*β*, yielding 100% in AUC values. Note that Projection method does not make any perturbation to the original kernel when positive semi-definite(GHI kernel when *α*=*β*), we can get conclusion that GHI kernel is a preferred kernel for tumor differentiation with NSCLC data. When *α* differs from *β*, different results are shown. When *α*=1,*β*=2, Projection method shows 99.72% in averaged AUC values with 0.01% standard deviation, while GHI kernel method only can get 64.07% in Averaged AUC values with a large standard deviation 7.42%. When *α*=2,*β*=3, Projection method shows 99.99% in averaged AUC values with 0 standard deviation, while GHI kernel method can get 73.07% in averaged AUC values with a large standard deviation 8.17%. Exceptions happen when *α*=1,*β*=3 where Projection method can only get 61.46% in averaged AUC values and GHI kernel method is even worse, achieving only 51.47% in averaged AUC values.

We can conclude that the performance of projection method is not always similar for different pairs of (*α*,*β*). There exists best (*α*,*β*) for inducing best projection method, but different data sets may be suitable to different pairs. GHI kernel method sometimes when kernel is indefinite can also perform well. But in general, projection method is clearly better than the GHI kernel for the above considered data sets.

#### Experiments on Cosine kernel

Table 5 compares the performance of Projection method with Cosine Kernel method for the considered datasets. Our Projection Method demonstrates visible better performance compared to Cosine Kernel method in terms of averaged AUC values. From the bold text on the left column of table, we can see that Projection method is superior for almost all the cases (except *q*=8 where the two methods show comparable performance with each other). Apart from that, the Projection method is more stable than Cosine Kernel method because the standard deviation of AUC values for each data set is smaller in Projection method. In Live Disorder Data set, the averaged AUC values of Cosine Kernel method is 65.63% with standard deviation 2.75%, and Projection method is much better than Cosine Kernel method, achieving 73.71% AUC values in average. The superiority of Projection method over Cosine Kernel method is clearly demonstrated in Sonar Data as well. The averaged AUC value for Cosine Kernel method is 67.46% but 89.57% for Projection method. For Cystic Fibrosis Data and Leukemia Data, Projection Method shows a general decrement in performance with the increment of q. However, in Cosine Kernel method there is no obvious correlation between the performance and the value of q within the data sets. In NSCLC Data set, both the Projection method and Cosine Kernel method show unsatisfying performance though Projection method is clearly better than the original Cosine Kernel method.

**Table 5:** Averaged AUC values (%) of projection method and Cosine kernel for the considered datasets

Dataset	Projection method	Cosine kernel
Live disorder data	**73.71** ± **1.21**	65.63 ± 2.75
Sonar data	**89.57** ± **1.37**	67.46 ± 4.32
Breast data	**99.37** ± **0.06**	97.99 ± 3.09
Cystic (*q*=1)	**79.25** ± **1.80**	76.89 ± 3.24
Cystic (*q*=2)	**80.55** ± **1.38**	79.80 ± 1.84
Cystic (*q*=3)	**78.27** ± **1.59**	70.10 ± 4.01
Cystic (*q*=4)	**73.24** ± **2.15**	58.52 ± 4.95
Cystic (*q*=5)	**64.38** ± **3.85**	52.13 ± 4.30
Cystic (*q*=6)	**69.26** ± **2.11**	60.72 ± 5.36
Cystic (*q*=7)	**64.6** ± **2.38**	58.54 ± 3.80
Cystic (*q*=8)	63.17 ± 2.89	63.66 ± 3.21
Cystic (*q*=9)	**54.21** ± **2.30**	43.05 ± 2.38
Leukemia (*q*=1)	**94.36** ± **0.43**	90.73 ± 1.94
Leukemia (*q*=2)	**94.38** ± **0.79**	69.45 ± 4.81
Leukemia (*q*=3)	**95.20** ± **0.49**	69.97 ± 6.58
Leukemia (*q*=4)	**94.73** ± **0.45**	73.33 ± 5.99
Leukemia (*q*=5)	**91.23** ± **0.44**	71.81 ± 9.62
Leukemia (*q*=6)	**93.19** ± **0.66**	79.08 ± 6.96
Leukemia (*q*=7)	**90.56** ± **1.25**	65.26 ± 6.90
Leukemia (*q*=8)	**87.81** ± **0.98**	58.31 ± 2.87
Leukemia (*q*=9)	**87.52** ± **1.20**	55.88 ± 3.82
NSCLC	**52.91** ± **4.45**	48.64 ± 5.30

Bold face represents best performance for the considered data sets in the compared two methods: Projection method and Cosine Kernel method, and no marks are made if two methods show comparable performance

## Discussion

Experimental results show that the Projection method is better or comparable with the compared kernel methods: GHI kernel and Cosine kernel. Despite the fact that GHI kernel and Cosine kernel when indefinite sometimes can yield good performance, Projection method still demonstrate comparable performance. The necessity of Projection transformation for the considered indefinite kernels is clearly demonstrated. Projection method when *λ*≥1 can transform an indefinite kernel into a PSD one. The optimal *λ* determination for Projection Method focusing on four different divergences is also considered. From the deduced optimal *λ*, we focus on the one with LogDet Divergence as it is more realistic.

In the following, we will conduct experiments on the considered data sets, to confirm if suggested optimal *λ* of the Projection Method can show optimal performance in various values of *λ*>0.

### Optimal *λ* in the projection method for sonar data

**Fig. 2:**
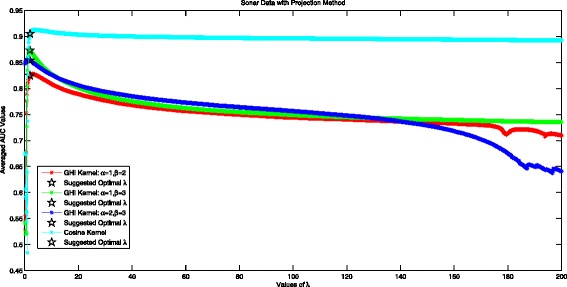
Averaged AUC values for different values of *λ* in projection method with two considered kernels using sonar data set

We set parameters *α*≠*β*∈{1,2,3} for GHI kernel and consider *λ*∈[0.1,200] with step size 0.1. Figure 2 plots the performance of Projection Method with different *λ*∈[0.1,200]. The ‘ ⋆’ shape in black color marks the performance of the Projection method with suggested optimal *λ* obtained under LogDet Divergence. The red line represents projected GHI kernel with *α*=1,*β*=2. The green line represents projected GHI kernel with *α*=1,*β*=3. The blue line represents projected GHI kernel with *α*=2,*β*=3. The cyan line plots the performance of projected Cosine Kernel.

The suggested optimal *λ* in Fig. 2 is 2.0 in Projected GHI kernel for all pairs of (*α*,*β*),*α*≠*β*. The performance of Projection Method shows a steady decrement when *λ*>2, implying that *λ*=2 is a good choice for projection method. When *λ*<1, the performance of projection method is quite unstable because the PSD property cannot be guaranteed.

It is very interesting to see that the suggested optimal *λ* is uniformly the same in the two considered kernels. Take projected GHI kernel with different (*α*,*β*) pairs for comparison, we can see that projected method with *α*=1,*β*=3 shows best performance, 0.8733, where the experimental best performance is shown to be 0.8735 achieving at *λ*=1.9. When *α*=1,*β*=2, the projection method with suggested optimal *λ* achieves 0.8246 in averaged AUC value, and the experimental best result is 0.8276. When *α*=2,*β*=3, the projection method with suggested optimal *λ* achieves 0.8540 in averaged AUC value, and the experimental best result is 0.8557. Considering the projected Cosine Kernel, the experimental best AUC value for Projected Cosine Kernel 0.9126 is achieved at *λ*=3.8, while our suggested optimal *λ*=2 yielding AUC value 0.9051, the difference between the two values is little: 0.0075. We can conclude that the suggested optimal *λ* can guarantee at least an near optimal performance.

### Optimal *λ* in the projection method for live disorder data

**Fig. 3:**
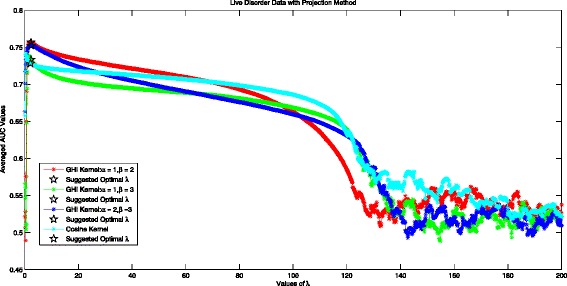
Averaged AUC values for different values of *λ* in projection method with two considered kernels using live disorder data set

Figure 3 shows the performance of Projection Method in different kernels for Live Disorder Data. The experimental optimal *λ* for GHI kernel with *α*=1,*β*=2 is 1.8, achieving averaged AUC value 0.7570. Our suggested *λ* under Logdet Divergence is 2.38, achieving averaged AUC value 0.7566. The performance difference in the Projection Method with theoretical optimal *λ*=2.38 and experimental optimal *λ*=1.8 is very small: 0.0004. When *α*=1,*β*=3, The experimental optimal *λ* for GHI kernel is 1.6, with the average AUC value equaling 0.7381. Our suggested optimal *λ* is 2.37, with averaged AUC value of 0.7333. The performance difference of the Projection Method with suggested optimal *λ*=2.37 and experimental optimal *λ*=1.6 is also very small: 0.0048. The experimental best AUC value of 0.7417 in Cosine kernel is achieved at *λ*=0.4, while our suggested optimal *λ*=2.17 yielding an AUC value of 0.7292. It can be seen that when *λ*>120, the performance of projected method with the two considered kernels fluctuates. When *λ*<120, the performance of projected method increases firstly and then decreases. The suggested optimal *λ* can guarantee at least an near optimal performance.

### Optimal *λ* in the projection method for breast cancer data

**Fig. 4:**
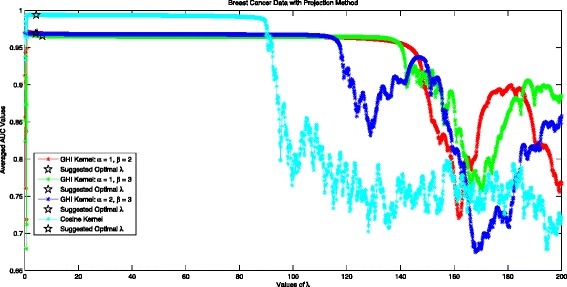
Averaged AUC values for different values of *λ* in projection method with two considered kernels using breast cancer data set

Figure 4 records the performance of the Projection Method for Breast Cancer Data. The experimental optimal *λ* is 1.1 for GHI kernel with *α*=1,*β*=2, achieving averaged AUC value of 0.9713. Our suggested optimal *λ* is 4.5957, with the averaged AUC value of 0.9693. The performance becomes slightly worse with increment of *λ*. The performance difference of the Projection Method with suggested optimal *λ*=4.5957 and experimental optimal *λ*=1.1 is subtle: 0.0018. Similar results are shown for GHI kernel with other (*α*,*β*) pairs. The experimental best AUC value 0.9941 is achieved at *λ*=0.8 for Cosine Kernel, while our suggested optimal *λ*=4.29 yielding AUC value 0.9939. Take projected GHI kernel and Cosine Kernel for comparison, we can see that projected cosine kernel shows visible better performance than projected GHI kernel, suggesting that we should choose projected Cosine kernel for breast cancer prediction. When all the kernels are considered, the suggested optimal *λ* is preferable in getting optimal performance for different values of *λ*.

### Optimal *λ* in the projection method for cystic fibrosis data

**Fig. 5:**
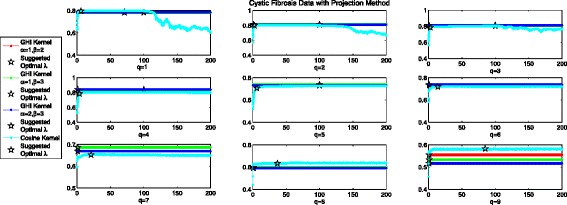
Averaged AUC values for different values of *λ* in projection method with two considered kernels using cystic fibrosis data set

Figure 5 records the performance of Projection method with the 2 considered kernels. We can see that in almost all cases the Projection method shows identical performance for *λ*∈(0,200] except for *q*=1 when *α*=1,*β*=2 and *α*=1,*β*=3. One possible explanation might be that GHI kernel is PSD already before projection (please see Additional file 1: Table SI). When *q*=1, *α*=1,*β*=2, the best AUC value when *λ*∈(0,200] is 0.7908, and the smallest AUC value is 0.7890, where theoretical optimal *λ* yields 0.7905 in AUC value which is near optimal. When *α*=1,*β*=3, the smallest AUC value is 0.7831 when *λ* approaching 200, the best AUC value is 0.7841 when *λ*=22.5, and the suggested optimal *λ* through Logdet Divergence yields 0.7840, which is also near optimal. Considering Cosine kernel, the performance of Projection method firstly improves and then descends gradually for *q*=1,2 and 3. For example, the best performance in experiment for q=1 is achieved at *λ*=77, with the AUC value 0.7987, while our suggested optimal *λ*=5.8 gets 0.7955 in AUC value, which is near optimal. When q increases, the performance of projection method improves firstly and stays relatively stable afterwards. For example, when q=4, suggested optimal *λ*=3.67 gets 0.7846 in AUC value and the experimental best performance 0.8012 is obtained when *λ*=26.7. It can be seen that denoising method when *λ*=1 achieves 0.6574 and flipping method when *λ*=2 achieves 0.7441, implying that projection method with suggested *λ* is better than these two methods. Although it is not optimal, the performance of projection method is satisfactory, which is slightly inferior to the optimal.

### Optimal *λ* in the projection method for leukemia data

**Fig. 6:**
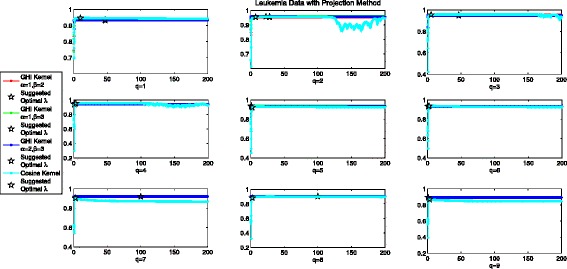
Averaged AUC values for different values of *λ* in projection method with two considered kernels using leukemia data set

Experimental Results for Projection Method with GHI kernel and Cosine kernel in leukemia data for *q*∈{1,2,…,9} are demonstrated in Fig. 6. Similar to Cystic Fibrosis Data, Projection method in GHI kernel shows almost identical performance for *λ*∈(0,200] for *q*∈{4,5,6,7,8,9}. This is consistent with the results in Table SI (Please refer to Additional file 1) where original GHI kernel is positive semi-definite when *q*≥4, as the minimal eigenvalues of the kernel matrix is 0. When *q*=1, the experimental best AUC value is 0.9364 for *α*=1,*β*=2, and our suggested optimal *λ* yields 0.9342. When *q*=2, the experimental best AUC value is 0.9545 for *α*=1,*β*=2 when *q*=2, and our suggested optimal *λ* yields 0.9542. When *q*=3, the experimental best AUC value is 0.9539 for *α*=1,*β*=2 when *q*=2, and our suggested optimal *λ* yields 0.9539. Experimental results are similar for other pairs of (*α*,*β*). Results for projected GHI kernel show that our suggested optimal *λ* can induce a near optimal projection method. In the case of Cosine kernel, we can get some information from the cyan line in Fig. 6. For all the considered *q*, there is no overall tendency when *λ*≤2, but the averaged AUC values will slowly decrease in a steady manner when the optimal performance is achieved. Some interesting phenomenon can be detected where projection method always shows poor performance when *λ*=1 (Denoising Method). For example, when *q*=2 the averaged AUC value of projection method is 0.6134 for *λ*=1, but 0.7450 for *λ*=0.9 and 0.9318 for *λ*=1.1. When *q*=3 the averaged AUC value of projection method is 0.4221 for *λ*=1, but 0.7784 for *λ*=0.9 and 0.9151 for *λ*=1.1. This probably can be explained that denoising strategy neglects some hidden information embedded in the negative eigenvalues and eigenvectors which is critical for describing the Leukemia Data. Regarding to the suggested optimal *λ* in projected Cosine Kernel, we can see that Projection method with the suggested optimal *λ* can always get at least near optimal performance for all *q*∈{1,2,…,9}.

### Optimal *λ* in the projection method with NSCLC data

**Fig. 7:**
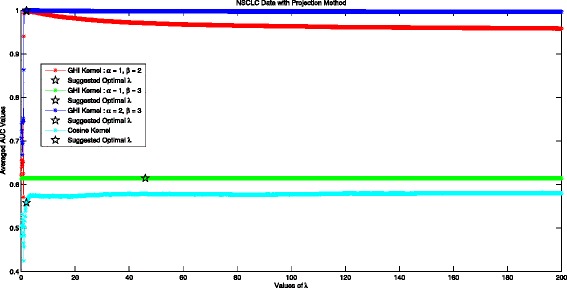
Averaged AUC values for different values of *λ* in projection method with two considered kernels using NSCLC data set

The performance of Projection Method with GHI kernel and Cosine Kernel in NSCLC Data is shown in Fig.7. When *α*=1,*β*=2, the optimal averaged AUC value in experiment is 0.9979 and projection method with our suggested optimal *λ*=2 can also ensure best performance 0.9979. When *α*=1,*β*=3, the experimental optimal averaged AUC value 0.6145 and projection method with *λ*=2 can also ensure best performance 0.6145. When *α*=2,*β*=3, the optimal averaged AUC value 1 in experiment and projection method with our suggested optimal *λ*=2 can also ensure equivalent best performance. Another conclusion can be made is that projection method with GHI kernel in different pairs of (*α*,*β*) may perform significantly different. In this experiment, we can see that *α*=1,*β*=3 is not fit for the task. Taking into consideration of the projected Cosine Kernel method, we can also conclude that cosine kernel is not suitable for dealing with tumor differentiation in NSCLC data.

Table 6 lists the optimal *λ* under Logdet Divergence with considered kernels for all the considered data sets. The first 3 columns refer to suggested optimal *λ* in Projected GHI kernel method. It is interesting to see that for cystic fibrosis data set, the suggested optimal *λ* is either 1 or 100 in most cases (except for *q*=1 when *α*=1,*β*=2 and *α*=1,*β*=3). The situation is similar for leukemia data set, where the suggested optimal *λ* in most cases is either 1 or 100 (*q*≥4). Note that our suggested optimal *λ* has the formula $ 1+\frac {m}{\sum _{i=1}^{m}d_{i} \text {tr}(K^{-1}\vec {p}_{i}\vec {p}_{i}')} $ (Please refer to Eq.(12)). Computational error may occur when the optimal *λ* is calculated to be close to ±*∞* which is not realistic. We therefore make the amendments accordingly where optimal *λ* is defined to be 1 when approaching −*∞* and 100 when approaching +*∞*. The last column lists the theoretical optimal *λ* for the considered data sets with Cosine kernel. Computational error does not have influence on Projection Method with Cosine Kernel in cystic fibrosis data as $\frac {m}{\sum _{i=1}^{m}d_{i} \text {tr}(K^{-1}\vec {p}_{i}\vec {p}_{i}')}$ is not close to 0. We can draw some conclusions from the table. Firstly, when kernels are different, the suggested optimal *λ* in most of the cases are different within the same data set. Secondly, when data sets are different, the suggested optimal *λ* in most of the cases are different even under the same kernel type. Focusing on the GHI kernel, we can see that the suggested optimal *λ* for different (*α*,*β*) differ from each other in most of the cases. Comparing GHI kernel and Cosine kernel, we can see that even for the same data set, it may happen that one type of kernel is positive semi-definite while the other type is indefinite, this can also partly explain why the suggested optimal *λ* is different.

**Table 6:** Optimal *λ* suggested in projection method with considered kernels

Methods	GHI Kernel			Cosine kernel
Dataset	*α*=1,*β*=2	*α*=1,*β*=3	*α*=2,*β*=3	
Live disorder data	2.38	2.37	2.45	2.17
Sonar data	2	2	2	2
Breast data	4.6	6.57	4.06	4.29
Cystic (*q*=1)	71	71	100	5.8
Cystic (*q*=2)	100	100	1	2.5
Cystic (*q*=3)	100	100	1	2.8
Cystic (*q*=4)	100	100	1	3.67
Cystic (*q*=5)	100	100	100	6.2
Cystic (*q*=6)	1	1	1	14
Cystic (*q*=7)	1	1	1	21
Cystic (*q*=8)	1	1	1	37
Cystic (*q*=9)	1	1	1	85
Leukemia (*q*=1)	47.33	47.33	46.67	10
Leukemia (*q*=2)	28.25	28.25	22.80	7.4
Leukemia (*q*=3)	46.5	46.5	47	5.42
Leukemia (*q*=4)	1	1	1	3.06
Leukemia (*q*=5)	1	1	1	2.33
Leukemia (*q*=6)	1	1	1	2.39
Leukemia (*q*=7)	100	100	100	2.56
Leukemia (*q*=8)	100	100	100	2.67
Leukemia (*q*=9)	1	1	1	2.98
NSCLC	2.0	46	2	2

### Any better optimal *λ* for projection method?

As stated above, we can see that under Logdet divergence, we can determine an near optimal *λ* for projection method. In this subsection, we are considering if there is any way to improve the projection method, in terms of finding a better optimal *λ*. Recall that Von Neumann divergence has the formula *D*_*ϕ*_(*K*,*K*_0_)=tr(*K* log*K*−*K* log*K*_0_−*K*+*K*_0_), we here did a little perturbation to the formula *D*_*ϕ*_(*K*,*K*_0_)=tr*K*tr(log*K*− log*K*_0_)+tr(−*K*+*K*_0_). Then we can determine optimal *λ* through minimizing the following function

$${\begin{aligned} V(\lambda)&=\left(\sum\limits_{i=1}^{m}(1-\lambda)d_{i}+\sum\limits_{i=m+1}^{n}d_{i}\right)\\ & \quad\times \left(\sum\limits_{i=1}^{m}(1 \,-\, \lambda)d_{i}\text{tr}\! \left(\! K_{0}^{-1}\vec{p}_{i}\vec{p}_{i}' \,+\, \sum\limits_{i=m+1}^{n}\! d_{i}\text{tr}(K_{0}^{-1}\vec{p}_{i}\vec{p}_{i}')\! \right)\! \right) \\ & \quad+\sum\limits_{i=1}^{m}d_{i} \end{aligned}} $$

We can easily get

$$\begin{array}{@{}rcl@{}} &V'(\lambda)=-2(1-\lambda)\sum\limits_{i=1}^{m}d_{i}\sum\limits_{i=1}^{m}\text{tr}(K_{0}^{-1}\vec{p}_{i}\vec{p}_{i}')\\ & \qquad\qquad\qquad - 2\sum_{i=1}^{m}d_{i}\times\sum\limits_{i=m+1}^{n}d_{i}\text{tr}(K_{0}^{-1}\vec{p}_{i}\vec{p}_{i}')+\sum\limits_{i=1}^{m}d_{i} \end{array} $$

Therefore, the new optimal *λ*_opt1_ is of the following formula:

$$\lambda_{\text{opt1}}=\frac{\sum_{i=1}^{n}d_{i}\text{tr}(K_{0}^{-1}\vec{p}_{i}\vec{p}_{i}')-0.5}{\sum_{i=1}^{m}d_{i}\text{tr}(K_{0}^{-1}\vec{p}_{i}\vec{p}_{i}')}. $$

We next conducted experiments on all the considered data sets to see the comparison of optimal *λ* from Logdet divergence *λ*_opt_ and the newly proposed *λ*_opt1_ in conjunction with projection method.

- Lambda Comparison with Projection Method in Sonar Data, Live Disorder Data, Breast Cancer Data and NSCLC Data
  As shown in the table (Table 7), we can see that the newly determined optimal *λ* through perturbed Von Neumann Divergence shows similar performance with the optimal *λ* generated by Logdet divergence. The only clear difference can be detected for Sonar data in GHI kernel when *α*=2,*β*=3 and Cosine Kernel. For GHI kernel *α*=2,*β*=3 we can see that *λ*_opt_ is superior to *λ*_opt1_, while for Cosine kernel, *λ*_opt1_ is superior to *λ*_opt_. Regarding the determined optimal *λ* under different divergences, we can see that *λ*_opt_ differs from *λ*_opt1_. For GHI kernel case, the determined optimal *λ* under Logdet Divergence and perturbed von-Neumann Divergence is similar to each other in Live data set but quite different in other data sets. For cosine kernel case, *λ*_opt_ and *λ*_opt1_ are quite different from each other. We can see that though the determined optimal *λ* under Logdet Divergence and perturbed von-Neumann Divergence is different, the performance is comparable. When we compare both kernels, we can see that Cosine kernel with *λ*_opt1_ is a preferred option.

**Table 7:** Optimal *λ* comparison in projection method with considered kernels in sonar data, live disorder data, breast cancer data and NSCLC data

		*α*=1,*β*=2	*α*=1,*β*=3	*α*=2,*β*=3	Cosine
Sonar	(*λ*_opt_,AUC_opt_)	(2.00,0.8266)	(2.00,0.8787)	*(2.00,0.8585)*	(2.00,0.9034)
	(*λ*_opt1_,AUC_opt1_)	(2.59,0.8284)	(2.16,0.8784)	(4.32,0.8486)	*(8.30,0.9118)*
Live	(*λ*_opt_,AUC_opt_)	(2.38,0.7559)	(2.37,0.7397)	(2.45,0.7543)	(2.17,0.7292)
	(*λ*_opt1_,AUC_opt1_)	(2.08,0.7571)	(2.04,0.7415)	(2.09,0.7542)	(6.70,0.7249)
Breast	(*λ*_opt_,AUC_opt_)	(4.60,0.9689)	(6.57,0.9659)	(4.06,0.9684)	(4.29,0.9937)
	(*λ*_opt1_,AUC_opt1_)	(2.03,0.9702)	(2.02,0.9675)	(2.20,0.9686)	(13.04,0.9936)
NSCLC	(*λ*_opt_,AUC_opt_)	(2.00,0.9996)	(2.00,0.9959)	(2.00,0.9903)	(2.00,0.4059)
	(*λ*_opt1_,AUC_opt1_)	(4.96,0.9990)	(3.58,0.9978)	(2.69,0.9910)	(2.30,0.4010)

The italicize represents visible difference detected for projection methods with different optimal *λ*
- Lambda Comparison with Projection Method in Cystic Fibrosis Data
  From Table 8, we can get some conclusions. For GHI kernel, it is obvious that projection method shows almost identical performance with *λ*_opt_ and *λ*_opt1_. It is interesting to see that in GHI kernel case, *λ*_opt_ and *λ*_opt1_ are equal to each other expect when *q*=1 for *α*=1,*β*=2 and *α*=1,*β*=3. From Table SI we know that GHI kernel in these 2 cases is indefinite. Although the values of *λ*_opt_ and *λ*_opt1_ are quite different from each other when *q*=1 for *α*=1,*β*=2 and *α*=1,*β*=3, the performances are similar to each other. When it comes to Cosine kernel, we can see that projection method with *λ*_opt1_ tends to perform better for *q*∈{1,2,3,4,5,6,9}. Clear differences can be detected when *q*=3,4,5 that are marked in bold face. Besides, *λ*_opt1_ in Cosine kernel is larger than *λ*_opt_ for most cases (*q*∈{1,2,…,7}), meaning that projection method with Cosine kernel tends to show better performance for relatively large *λ*. When we compare GHI kernel and Cosine kernel, we find that GHI kernel in general tends to show better performance for small *q*, and Cosine kernel shows better performance when *q* is large.

**Table 8:** Optimal *λ* comparison in projection method with considered kernels in cystic fibrosis data

		*α*=1,*β*=2	*α*=1,*β*=3	*α*=2,*β*=3	Cosine
*q*=1	(*λ*_opt_,AUC_opt_)	(71,0.7771)	(71,0.7711)	(100,0.7829)	(5.8,0.7889)
	(*λ*_opt1_,AUC_opt1_)	(36.5,0.7775)	(36.5,0.7713)	(100,0.7829)	(28.3,0.7912)
*q*=2	(*λ*_opt_,AUC_opt_)	(100,0.8031)	(100,0.8114)	(1,0.8209)	(2.5,0.7951)
	(*λ*_opt1_,AUC_opt1_)	(100,0.8031)	(100,0.8114)	(1,0.8209)	(43.38,0.7959)
*q*=3	(*λ*_opt_,AUC_opt_)	(100,0.8103)	(100,0.8140))	(1,0.8033)	(2.8,0.7978)
	(*λ*_opt1_,AUC_opt1_)	(100,0.8103)	(100,0.8140)	(1,0.8033)	*(34.3,0.8111)*
*q*=4	(*λ*_opt_,AUC_opt_)	(100,0.8296)	(100,0.8356)	(1,0.8286)	(3.67,0.7825)
	(*λ*_opt1_,AUC_opt1_)	(100,0.8296)	(100,0.8356)	(1,0.8286)	*(26.58,0.7979)*
*q*=5	(*λ*_opt_,AUC_opt_)	(100,0.7400)	(100,0.7272)	(100,0.7405)	(6.2,0.6973)
	(*λ*_opt1_,AUC_opt1_)	(100,0.7400)	(100,0.7272)	(100,0.7405)	*(27,0.7137)*
*q*=6	(*λ*_opt_,AUC_opt_)	(1,0.7173)	(1,0.7164)	(1,0.7224)	(14,0.7144)
	(*λ*_opt1_,AUC_opt1_)	(1,0.7173)	(1,0.7164)	(1,0.7224)	(34.16,0.7156)
*q*=7	(*λ*_opt_,AUC_opt_)	(1,0.6702)	(1,0.6721)	(1,0.6713)	(21,0.6620)
	(*λ*_opt1_,AUC_opt1_)	(1,0.6702)	(1,0.6721)	(1,0.6713)	(22.17,0.6616)
*q*=8	(*λ*_opt_,AUC_opt_)	(1,0.5928)	(1,0.5791)	(1,0.5935)	(37,0.6388)
	(*λ*_opt1_,AUC_opt1_)	(1,0.5928)	(1,0.5791)	(1,0.5935)	(19.25,0.6387)
*q*=9	(*λ*_opt_,AUC_opt_)	(1,0.5146)	(1,0.5107)	(1,0.5254)	(85,0.5637)
	(*λ*_opt1_,AUC_opt1_)	(1,0.5146)	(1,0.5107)	(1,0.5254)	(17.5,0.5688)

The italicize represents visible difference detected for projection methods with different optimal *λ*
- Lambda Comparison with Projection Method in Leukemia Data
  We can get similar conclusions for Leukemia data. As shown in the table (Table 9), projection method shows almost identical performance with *λ*_opt_ and *λ*_opt1_ though different optimal *λ* values are obtained (Please check *q*=1,2,3 respectively). When *q*=1, *λ*_opt1_ is smaller than *λ*_opt_. When *q*=2,3 respectively, *λ*_opt1_ is larger than *λ*_opt_. Though values of optimal *λ* differ from each other, the performances are quite similar, meaning that projection method with GHI kernel for Leukemia data is less sensitive in the optimal *λ*. When *q*∈{4,5,6,7,8,9}, *λ*_opt_ and *λ*_opt1_ are identical, we can see from Table SI that GHI kernel in these cases are PSD already. For Cosine Kernel, optimal *λ* determined by Logdet Divergence and perturbed Von-Neumann Divergence differs. Projection method with *λ*_opt1_ performs slightly better than projection method with *λ*_opt_. Besides, *λ*_opt1_ in Cosine kernel is larger than *λ*_opt_, implying that projection method tends to show better performance for large *λ*. When we focus on the performance of projection method with *λ*_opt1_, we can find that different from Cystic Fibrosis data set, the performance of projected cosine kernel with *λ*_opt1_ tends to show better performance for small *q* while projected GHI kernel with *λ*_opt_ tends to show better performance for large *q*.

**Table 9:** Optimal *λ* comparison in projection method with considered kernels in leukemia data

		*α*=1,*β*=2	*α*=1,*β*=3	*α*=2,*β*=3	Cosine
*q*=1	(*λ*_opt_,AUC_opt_)	(47.3,0.9418)	(47.3,0.9377)	(46.7,0.9365)	(10,0.9469)
	(*λ*_opt1_,AUC_opt1_)	(26.8,0.9419)	(26.8,0.9377)	(27.8,0.9367)	(16.6,0.9472)
*q*=2	(*λ*_opt_,AUC_opt_)	(28.3,0.9551)	((28.3,0.9551)	(22.8,0.9582)	(7.4,0.9541)
	(*λ*_opt1_,AUC_opt1_)	(35.9,0.9550)	(35.9,0.9551)	(29.3,0.9582)	(24.7,0.9555)
*q*=3	(*λ*_opt_,AUC_opt_)	(46.5,0.9512)	(46.5,0.9540)	(47,0.9500)	(5.42,0.9573)
	(*λ*_opt1_,AUC_opt1_)	(87.8,0.9512)	(87.8,0.9551)	(88.8,0.9500)	(23.1,0.9593)
*q*=4	(*λ*_opt_,AUC_opt_)	(1,0.9427)	(1,0.9416)	(1,0.9405)	(3.06,0.9485)
	(*λ*_opt1_,AUC_opt1_)	(1,0.9427)	(1,0.9416)	(1,0.9405)	(18.6,0.9522)
*q*=5	(*λ*_opt_,AUC_opt_)	(1,0.9352)	(1,0.9362)	(1,0.9363)	(2.33,0.9175)
	(*λ*_opt1_,AUC_opt1_)	(1,0.9352)	(1,0.9362)	(1,0.9363)	(11.08,0.9259)
*q*=6	(*λ*_opt_,AUC_opt_)	(1,0.9310)	(1,0.9319)	(1,0.9311)	(2.39,0.9333)
	(*λ*_opt1_,AUC_opt1_)	(1,0.9310)	(1,0.9319)	(1,0.9311)	(7.74,0.9337)
*q*=7	(*λ*_opt_,AUC_opt_)	(100,0.9201)	(100,0.9236)	(1,0.9212)	(2.56,0.8993)
	(*λ*_opt1_,AUC_opt1_)	(100,0.9201)	(100,0.9236)	(1,0.9212)	(5.03,0.8921)
*q*=8	(*λ*_opt_,AUC_opt_)	(100,0.9035)	(100,0.9096)	(100,0.9059)	(2.67,0.8795)
	(*λ*_opt1_,AUC_opt1_)	(100,0.9035)	(100,0.9096)	(100,0.9059)	(3.56,0.8845)
*q*=9	(*λ*_opt_,AUC_opt_)	(1,0.8936)	(1,0.8915)	(1,0.8899)	(2.98,0.8734)
	(*λ*_opt1_,AUC_opt1_)	(1,0.8936)	(1,0.8915)	(1,0.8899)	(3.72,0.8735)

The boldface represents best performance detected for projection methods with different optimal *λ*, and no marks are made if two methods show comparable performance

In summary, when *λ*∈(0,1), the positive semi-definiteness of the projected kernel matrix cannot be assured, and the performance tends to be extremely unstable. The suggested optimal *λ* in Projection method is related to the eigenvalues in original kernel matrix, and thus varies in different data sets. Besides, the suggested optimal *λ* under Logdet Divergence and perturbed Von-Neumann Divergence differs from each other in the same data sets in most cases. Even in that case, projection method under the two different cases can still guarantee near optimal performance. It can be seen that when optimal *λ* under Logdet Divergence and optimal *λ* under perturbed Von-Neumann Divergence is very different, the performance of projection method in both cases is still similar, showing that in this case projection method is relatively insensitive to the values of suggested optimal *λ* (projection method with a large range of *λ* values can suggest near optimal performance). Our suggested theoretical *λ* under Logdet Divergence and perturbed Von-Neumann Divergence sometimes cannot guarantee the best performance. There are two possible reasons. One possible reason is that the optimal *λ* determination by unconstrained optimization in framework of kernel learning hypothesized the positive definiteness of the kernels, but we use indefinite kernels in this case. Another possible reason is that the inverse of kernel was substituted by pseudo inverse.

## Conclusions

In this paper, we propose projection method for addressing indefinite kernel learning problems. The projection method is construed from an eigen-space perspective. It is very flexible by varying the parameter *λ*, to change from the denoising method to the flipping method. These two spectrum based methods are well-known techniques in dealing with indefinite kernels. Two kernels that are not generally PSD are introduced for comparison: GHI kernel method and the Cosine kernel method. We show better performance for projection method in terms of AUC values under 5-fold cross-validations. The optimal *λ* embedded in the Projection Method can be determined by solving an unconstrained optimization problem. Experimental studies show consistence with theoretical analysis as projection method with our suggested *λ* can always guarantee at least near optimal performance for *λ*>0. In the pursuit of precise optimal *λ* determination method, we also compared optimal *λ* determination with Logdet Divergence and perturbed Von-Neumann Divergence, aiming at finding better *λ* in projection method. The determined optimal *λ* differs from each other for different kernels and data sets involved, and the results obtained are in general similar. Our proposed projection method may be regarded as a good choice for dealing with indefinite kernels. Future work may contribute to the development of more precise optimal *λ* determination method and the development of more variants of projection method for indefinite kernels, hoping to be applied in other areas.

## Additional file

Additional file 1 Table SI. Additional file 1 contains one table. The table SI records Maximum and Minimum Eigenvalues with Considered Kernels generated for all the considered Data sets. (PDF 14 kb)
