# Supplementary material for: Optimal projection method determination by Logdet Divergence and perturbed von-Neumann Divergence
Source: BMC Syst Biol. 2017 Dec 14;11(Suppl 6):115. doi: 10.1186/s12918-017-0479-0 (PMC5751553; doi:10.1186/s12918-017-0479-0)
Supplement: Additional file 1 — Table SI. Additional file 1 contains one table. The table SI records Maximum and Minimum Eigenvalues with Considered Kernels generated for all the considered Data sets. (PDF 14 kb) [file 12918_2017_479_MOESM1_ESM.pdf]

Table SI: Maximum and Minimum Eigenvalues with Considered Kernels in considered Datasets

Methods / Data	GHI Kernel $\alpha=1,\beta=1$	$\alpha=1,\beta=2$	$\alpha=1,\beta=3$	$\alpha=2,\beta=2$	$\alpha=2,\beta=3$	$\alpha=3,\beta=3$	Cosine Kernel
Live Disorder	$1\times10^5(0)$	1×10^5(-2.3)	$1\times10^5(-4.1)$	$1\times10^9(0)$	1×10^9(-1.2)	$1\times10^{13}(0)$	243.8(-28)
Sonar	$1\times10^4(0)$	1×10^4(-2.3)	1×10^4(-5.1)	$1\times10^8(0)$	1×10^8(-0.8)	$1\times10^{12}(0)$	39.5(-110)
Breast	$4.6\times10^3(0)$	4.2×10^3(-5.3)	3.9×10^3(-6.4)	$3.9\times10^3(0)$	$3.7\times10^3(-1)$	$3.5\times10^3(0)$	432.8(-253.5)
Cystic ($q=1$)	498.4(0)	498.4(-0.08)	498.4(-0.08)	561.9(0)	561.9(0)	759.6(0)	35.6(-111)
Cystic ($q=2$)	303.8(0)	303.8(0)	303.8(0)	315.1(0)	315.1(0)	347.5(0)	30.2(-125.2)
Cystic ($q=3$)	196.1(0)	196.1(0)	196.1(0)	196.8(0)	196.8(0)	198.7(0)	32.2(-131.5)
Cystic ($q=4$)	102.3(0)	102.3(0)	102.3(0)	102.4(0)	102.4(0)	102.6(0)	52.7(-119.4)
Cystic ($q=5$)	47.3(0)	47.3(0)	47.3(0)	47.3(0)	47.3(0)	47.3(0)	77.2(-94)
Cystic ($q=6$)	32.5(0)	32.5(0)	32.5(0)	32.5(0)	32.5(0)	32.5(0)	104.4(-65.4)
Cystic ($q=7$)	30.3(0)	30.3(0)	30.3(0)	30.3(0)	30.3(0)	30.3(0)	129.4(-38.5)
Cystic ($q=8$)	25.0(0)	25.0(0)	25.0(0)	25.0(0)	25.0(0)	25.0(0)	149.6(-38.5)
Cystic ($q=9$)	18.0(0)	18.0(0)	18.0(0)	18.0(0)	18.0(0)	18.0(0)	165.8(-5.8)
Leukemia ($q=1$)	$2.3\times10^3(0)$	$2.4\times10^3(-14.0)$	$2.4\times10^3(-14.0)$	$5.6\times10^3(0)$	$6.3\times10^3(-151.4)$	$1.7\times10^4(0)$	114.9(-178.8)
Leukemia ($q=2$)	$2.0\times10^3(0)$	$2.0\times10^3(-4.1)$	$2.0\times10^3(-4.1)$	$3.5\times10^3(0)$	$3.9\times10^3(-55.7)$	$8.3\times10^3(0)$	80.8(-222.4)
Leukemia ($q=3$)	$2.0\times10^3(0)$	$2.0\times10^3(-4.0)$	$2.0\times10^3(-4.0)$	$2.5\times10^3(0)$	$2.6\times10^3(-16.7)$	$3.5\times10^3(0)$	57.6(-188.8)
Leukemia ($q=4$)	$2.3\times10^3(0)$	$2.3\times10^3(0)$	$2.3\times10^3(0)$	$2.4\times10^3(0)$	$2.4\times10^3(0)$	$2.4\times10^3(0)$	67.9(-178.9)
Leukemia ($q=5$)	$2.8\times10^3(0)$	$2.8\times10^3(0)$	$2.8\times10^3(0)$	$2.8\times10^3(0)$	$2.8\times10^3(0)$	$2.8\times10^3(0)$	120.7(-188.4)
Leukemia ($q=6$)	$3.2\times10^3(0)$	$3.2\times10^3(0)$	$3.2\times10^3(0)$	$3.2\times10^3(0)$	$3.2\times10^3(0)$	$3.2\times10^3(0)$	120.6(-157.0)
Leukemia ($q=7$)	$3.4\times10^3(0)$	$3.4\times10^3(0)$	$3.4\times10^3(0)$	$3.4\times10^3(0)$	$3.4\times10^3(0)$	$3.4\times10^3(0)$	180.2(-170.0)
Leukemia ($q=8$)	$3.4\times10^3(0)$	$3.4\times10^3(0)$	$3.4\times10^3(0)$	$3.4\times10^3(0)$	$3.4\times10^3(0)$	$3.4\times10^3(0)$)	169.2(-146.2)
Leukemia ($q=9$)	$3.1\times10^3(0)$	$3.1\times10^3(0)$	$3.1\times10^3(0)$	$3.1\times10^3(0)$	$3.1\times10^3(0)$	$3.1\times10^3(0)$	198.6(-131.7)
NSCLC	$2.5\times10^7(0)$	$2.7\times10^7(-883.8)$	$2.7\times10^7(-877.3)$	$1.5\times10^8(0)$	$1.6\times10^8(-1.4\times10^4)$	$1.0\times10^9(0)$	13.9(-11.8)
